# The structure of natively iodinated bovine thyroglobulin

**DOI:** 10.1107/S2059798321010056

**Published:** 2021-10-29

**Authors:** Kookjoo Kim, Mykhailo Kopylov, Daija Bobe, Kotaro Kelley, Edward T. Eng, Peter Arvan, Oliver B. Clarke

**Affiliations:** a Department of Physiology and Cellular Biophysics, Columbia University Irving Medical Center, 1150 Saint Nicholas Avenue, New York, NY 10032, USA; b The National Resource of Automated Molecular Microscopy, Simons Electron Microscopy Center, New York Structural Biology Center, 89 Convent Avenue, New York, NY 10027, USA; c The Division of Metabolism, Endocrinology and Diabetes, University of Michigan Medical Center, Ann Arbor, MI 48105, USA

**Keywords:** cryoEM, thyroglobulin, thyroid hormone synthesis, thyroxine

## Abstract

Thyroglobulin is a homodimeric glycoprotein that is essential for the generation of thyroid hormones in vertebrates. Upon secretion into the lumen of follicles in the thyroid gland, tyrosine residues within the protein become iodinated to produce monoiodotyrosine (MIT) and diiodotyrosine (DIT). A subset of evolutionarily conserved pairs of DIT (and MIT) residues can then engage in oxidative coupling reactions that yield either thyroxine (T_4_; produced from coupling of a DIT ‘acceptor’ with a DIT ‘donor’) or triiodothyronine (T_3_; produced from coupling of a DIT acceptor with an MIT donor). Although multiple iodotyrosine residues have been identified as potential donors and acceptors, the specificity and structural context of the pairings (*i.e.* which donor is paired with which acceptor) have remained unclear. Here, single-particle cryogenic electron microscopy (cryoEM) was used to generate a high-resolution reconstruction of bovine thyroglobulin (2.3 Å resolution in the core region and 2.6 Å overall), allowing the structural characterization of two post-reaction acceptor–donor pairs as well as tyrosine residues modified as MIT and DIT. A substantial spatial separation between donor Tyr149 and acceptor Tyr24 was observed, suggesting that for thyroxine synthesis significant peptide motion is required for coupling at the evolutionarily conserved thyroglobulin amino-terminus.

## Abstract 2

Thyroglobulin is the precursor protein that serves as a substrate for thyroid hormone synthesis. Here, a three-dimensional cryoEM reconstruction of bovine thyroglobulin at an overall resolution of 2.6 Å is presented. The structure facilitates the definitive identification of thyroxine (acceptor) and dehydroalanine (donor) sites at two of the most evolutionarily conserved sites of hormone formation in vertebrates.

## 1. Introduction   

Thyroglobulin (Tg) is a 660 kDa (2 × 330 kDa) homodimeric protein which is the most highly expressed protein in the thyroid gland. The protein is secreted by thyrocytes into the thyroid follicle lumen where, upon iodination by thyroid peroxidase, Tg functions as a scaffold protein for thyroid hormonogenesis and as a storage protein for both thyroid hormones and iodide (van de Graaf *et al.*, 2001 ▸). Tg from the thyroid gland is the original source of the (iodine-containing) thyroid hormone thyroxine (T_4_), as well as some triiodo­thyronine (T_3_). T_4_ is the predominant form of the hormone released from the thyroid gland to the bloodstream; free T_4_ is derived from the proteolytic processing of iodinated Tg. Defects in Tg cause decreased hormone synthesis, leading to hypothyroidism, as well as feedback stimulation of the thyroid gland that can promote the development of goiter (Medeiros-Neto *et al.*, 1993 ▸; Zimmermann, 2009 ▸). T_3_, the more active form of the thyroid hormone, is largely generated by the deiodination of T_4_, which occurs to a considerable extent in nonthyroidal tissues (Dunn & Dunn, 1999 ▸; Fekete & Lechan, 2014 ▸).

Various tyrosine residues in the Tg molecule can be iodinated, but only a small number of T_4_ molecules are synthesized per molecule of Tg under iodine-sufficient conditions (Lamas *et al.*, 1989 ▸; Dupuy *et al.*, 1999 ▸). An oxidative coupling reaction in which a diiodotyrosine (DIT) acceptor receives (via a covalent quinol-ether linkage) the iodophenolic ring of a neighboring DIT donor results in the formation of thyroxine at an acceptor site and dehydroalanine at the corresponding donor site. While these features have long been understood, until recently the structure of Tg was unknown. Tg is a heavily glycosylated protein, with up to 20 potential N-glycosylation sites and one site of O-linked glycosylation in human Tg (Yang *et al.*, 1996 ▸). Heterogeneity of post-translational modifications (for example glycosylation and iodination) and conformational flexibility prevented structural characterization using X-ray crystallography. The recently reported cryoEM reconstruction of human Tg has provided a crucial breakthrough (Coscia *et al.*, 2020 ▸). However, in spite of this, basic questions remain unanswered regarding the structural mechanism of thyroid hormonogenesis. In particular, the human Tg reconstruction did not exhibit density features corresponding to iodinated or thyroxinated tyrosines, leaving the structural mechanism of hormonogenesis, and the structural context of the post-reaction thyroxinated state, to be revealed.

In this report, we present a high-resolution structure of purified Tg (iodinated *in vivo* with normal iodide content) from the bovine thyroid gland. We have been able to definitively identify three (of 70) tyrosine side chains per Tg monomer that are modified either to diiodotyrosine (Tyr2041) or thyroxine (Tyr24 and Tyr2575, indicative of acceptor sites). In addition, we observe four tyrosines with no discernible side chain despite well defined main-chain density, which we have tentatively assigned as dehydroalanines (Tyr89, Tyr149, Tyr1395 and Tyr2542); these are the only identifiable candidates as donors in thyroid hormone formation. We also provide structural evidence to suggest that the peptides containing acceptor and donor DIT residues are mobile prior to coupling and that the acceptor peptide is immobilized after T_4_ is formed and is sequestered within a thyroxine-binding pocket.

## 2. Methods   

### 2.1. Thyroglobulin sample preparation   

All steps were performed on ice unless stated otherwise. 10 mg of bovine thyroglobulin powder (∼0.6% iodine reported for Sigma–Aldrich catalog No. T9145) was added to 600 µl buffer consisting of 10 m*M* HEPES pH 7.4, 100 m*M* NaCl. The sample was thoroughly mixed and solubilized, and was filtered through a 0.22 µm cellulose acetate centrifuge tube filter (Corning) before loading onto a TSKgel BioAssist G4SWxl column (Tosoh Bioscience) pre-equilibrated with gel-filtration buffer (10 m*M* HEPES pH 7.4, 100 m*M* NaCl). Purified Tg was eluted at 0.5 ml min^−1^ and the fractions containing Tg were combined and concentrated on a 100 kDa cutoff centrifugation filter (Millipore) to 7 mg ml^−1^ as determined using spectroscopy (absorbance at 280 nm). Upon screening the first set of grids, we observed that most of the Tg molecules were dissociated into low-molecular-weight components, although a chromatogram from size-exclusion chromatography of a solubilized Tg sample showed a single Gaussian peak, and purity was further confirmed by SDS–PAGE (Supplementary Fig. S1). We hypothesized that the Tg molecules were adsorbed to the air–water interface and theorized that a small addition of a detergent with high critical micelle concentration (CMC) would protect the Tg molecules from denaturation, as reported in other studies (Noble *et al.*, 2018 ▸; Chen *et al.*, 2019 ▸). Thus, we added 0.2%(*w*/*v*) CHAPS (CMC of ∼0.49%; GoldBio) to the purified Tg sample after concentration. We observed that the addition of CHAPS greatly improved the quality of the micrographs (Supplementary Fig. S1*c* ). Cryogenic electron tomography (cryoET) data collected from Tg grids with or without the addition of CHAPS further confirmed that the Tg particles are protected from denaturation and are away from the air–water interface when 0.2% CHAPS was added to the sample before vitrification of the sample on the grids (Supplementary Movies S1 and S2). Briefly, the grids were subjected to tomographic data collection with stage tilt starting from −45° to +45° in 3° increments on a Titan Krios microscope (FEI) operated at 300 kV and equipped with an energy filter (slit width 20 eV) and a K2 direct electron detector (Gatan) at a pixel size of 2.798 Å with a total electron dose of 143 e^−^ Å^−2^. Frames were aligned with *MotionCor*2 (Zheng *et al.*, 2017 ▸) and tilt-series alignment was performed with *Protomo* implemented in the *Leginon*/*Appion* software package (Noble & Stagg, 2015 ▸; Suloway *et al.*, 2005 ▸; Lander *et al.*, 2009 ▸).

### 2.2. CryoEM grid preparation and data collection   

UltrAuFoil R0.6/1 holey gold grids (Quantifoil) were prepared by plasma cleaning with a mixture of H_2_ and O_2_ (Gatan). 3 µl of the Tg sample was applied to the plasma-cleaned grids. The grids were blotted for 7–9.5 s and vitrified by plunge-cooling in liquid ethane (Dubochet *et al.*, 1988 ▸; Wagenknecht *et al.*, 1988 ▸) with a Vitrobot (FEI) operated at 4°C, 100% humidity, blot force 3, waiting time 30 s and drain time 0 s. Grids were screened with a Tecnai F20 microscope (FEI) equipped with a K2 Summit direct electron detector (Gatan) operated at 200 kV. Microscope operation and data collection were carried out using the *Leginon* and *Appion* software (Suloway *et al.*, 2005 ▸; Lander *et al.*, 2009 ▸).

The screened grids were then subjected to high-resolution cryoEM data acquisition (Supplementary Fig. S1*d* ) on a Titan Krios microscope (FEI) equipped with an energy filter (slit width 20 eV) and a K2 direct electron detector (Gatan) operated at 300 kV. 6772 micrographs were collected at a nominal magnification of 130 000× in electron-counting mode, corresponding to a calibrated pixel size of 1.06 Å on the specimen scale. The electron dose rate on the camera was set to 8 e^−^ per pixel per second, and 10 s image stacks with 50 frames (200 ms per frame; 1.43 e^−^ Å^−2^ per frame) were collected.

### 2.3. Data processing   

The data-processing workflow is summarized in Supplementary Fig. S2. Movie frames of 6772 micrographs were aligned using the *MotionCor*2 algorithm as implemented in *RELION*3 (Zheng *et al.*, 2017 ▸; Zivanov *et al.*, 2018 ▸) with 5 × 5 patches with a *B* factor of 150 Å^2^. CTF estimation was performed for the motion-corrected averages using *CTFFIND*4 (Rohou & Grigorieff, 2015 ▸). Micrographs with an estimated resolution of worse than 5 Å were removed, resulting in a set of 5016 micrographs.

Thyroglobulin particles were manually picked from the motion-corrected micrographs to generate 2D templates for further template-based automatic particle picking using *relion_autopick*. The initial set of 1 176 329 template-picked particles were extracted from the micrographs and imported into *cryoSPARC* (Punjani *et al.*, 2017 ▸) for 2D classification, *ab initio* reconstruction and heterogeneous refinement, which resulted in 539 260 particles after the removal of poor-quality particles and nonprotein contaminants. Subsequent homogeneous refinement of the selected particles resulted in a 3.01 Å resolution reconstruction with no symmetry imposed. Per-particle defocus refinement and beam-tilt refinement grouped by image shift as estimated from *Appion* metadata and Bayesian polishing were executed in *RELION*3 (Zivanov *et al.*, 2019 ▸). Subsequent refinement using the 3D auto-refine routine in *RELION*3 resulted in a reconstruction with a global resolution of 2.7 Å. Thyroglobulin displays substantial conformational flexibility, both between the halves of the dimer and between the core and the arm regions of the molecule. In particular, the local resolution around the arm region of the Tg molecule was estimated to be ∼4 Å, with anisotropic density features (Supplementary Fig. S3*a* ), making model building in this region challenging. To improve the resolution around the core and the arm regions of Tg, focused refinements of these regions were performed in *RELION* after *C*2 symmetry expansion of the particle set. The resulting maps from the focused refinements were then combined after real-space alignment with the global reconstruction by taking the maximum value at each voxel of the pair of maps (Supplementary Figs. S2 and S3). Focused refinement of a Tg monomer including the arm region gave a reconstruction at 2.61 Å, and calculation of Fourier shell correlation (FSC) with a mask surrounding the carboxy-terminal cholinesterase-like (ChEL) domain, which comprises the core region of Tg, yielded a resolution of 2.3 Å, with high-resolution features consistent with this value, including clearly defined carbonyl densities and water molecules in the combined map (Supplementary Fig. S3*b* ). To improve the overall quality of the combined EM map and the local quality of the map around T_4_ binding sites, density-modified maps of focused refinements on the region surrounding half of the dimeric Tg molecule and the arm region were generated using *phenix.resolve_cryo_em* (Terwilliger *et al.*, 2020 ▸), which is included in the *Phenix* software suite (Liebschner *et al.*, 2019 ▸). The resulting density-modified maps were then combined after real-space alignment with the global reconstruction in *UCSF Chimera*, as outlined above. To improve the local quality of the map around the arm region, a modified map for this region was generated using *DeepEMhancer* (Sanchez-Garcia *et al.*, 2020 ▸). The *DeepEMhancer*-modified map displayed enhanced connectivity and side-chain resolution in some poorly resolved areas, which assisted in the final stages of model building.

An atomic model of the protein was built in *Coot* (Emsley *et al.*, 2010 ▸) starting from a homology model of the ChEL domain (residues ∼2200–2720), using the structure of butyrylcholinesterase (PDB entry 6emi; Brazzolotto *et al.*, 2017 ▸) as a template. A representative model and density fit is depicted in Supplementary Fig. S4. Upon closer inspection of the model and the map, the primary amino-acid sequence of the protein was not consistent with the representative bovine thyro­globulin sequence entry in UniProt (UniProt P01267). After inspecting the map and comparison with other available bovine Tg sequences, we identified UniProt A0A4W2CHS8 as the correct sequence, as it matches the 3D reconstruction of our bovine Tg sample (Supplementary Fig. S5). Model and density visualizations were prepared using *UCSF Chimera* (Pettersen *et al.*, 2004 ▸) and *ChimeraX* (Goddard *et al.*, 2018 ▸). Data-collection and model-refinement statistics are summarized in Table 1 ▸. The structure of the bovine Tg molecule has been deposited in the PDB (PDB entry 7n4y). Combined EM maps, a global reconstruction map, focused refined maps and local masks around the arm region of the Tg monomer and the region surrounding half of the dimeric Tg molecule, and their corresponding half-maps, have been deposited in the EMDB (EMDB entry EMD-24181). The raw movie files have been deposited in EMPIAR (EMPIAR entry EMPIAR-10833).

## 3. Results   

### 3.1. Domain arrangement and architecture of Tg   

**Figure 1:**
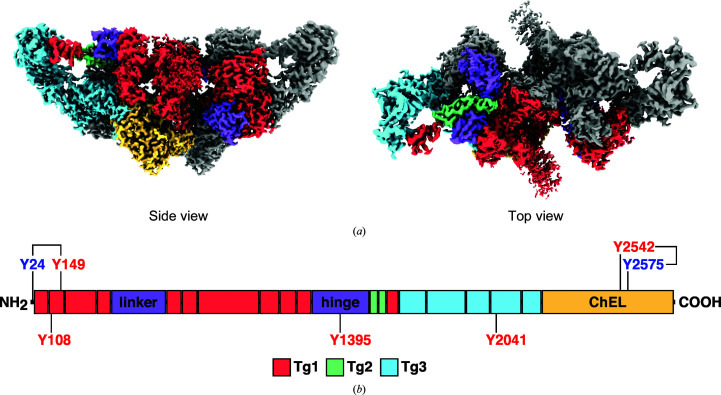
CryoEM map and architecture of bovine Tg. (*a*) CryoEM map of bovine Tg. One monomer is colored by domain, while the other is shown in gray. The dimerization interface diagonally splits the Tg homodimer. Tg1 domains are colored red, Tg2 domains are colored mint green, Tg3 domains are colored aqua, linker and hinge domains are colored purple and the ChEL domain is colored yellow. The map was split in *UCSF ChimeraX* to show the disordered handle of the Tg dimer by applying different contour levels to the split maps (map contour levels 0.03 and 0.04). (*b*) Schematic representation of the domain organization of the Tg monomer. Modified tyrosine residues are noted. The acceptor tyrosine residues Tyr24 and Tyr2575 were thyroxinated. The donor tyrosine residues Tyr108, Tyr149, Tyr1395 and Tyr2542 were missing side chains, hinting at a modification to dehydroalanine. Tyr2041 was modified to diiodotyrosine (DIT) (Supplementary Fig. S6).

**Figure 2:**
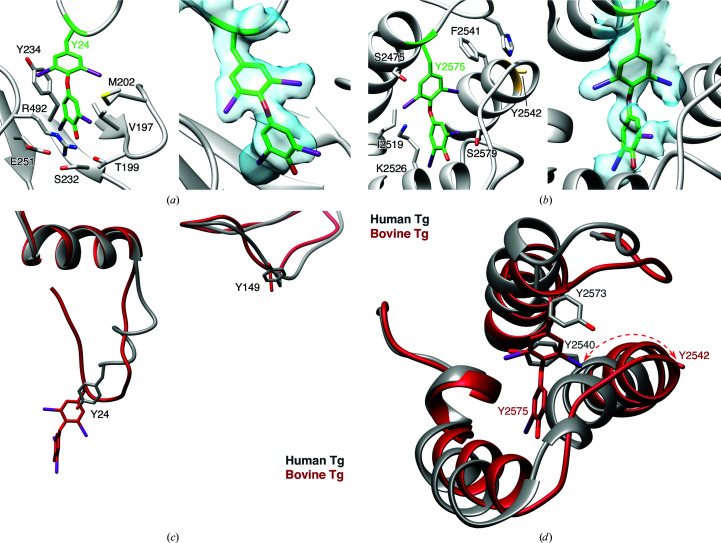
Iodotyrosines in bovine Tg. Side chains and cryoEM map density fit for thyroxinated side chains at Tyr24 (*a*) and Tyr2575 (*b*). Map contour level 0.032. (*c*) Compared with the human Tg structure (gray; PDB entry 6scj; Coscia *et al.*, 2020 ▸) with non-iodinated tyrosine side chains at Tyr2573 and Tyr2540 (Tyr2575 and Tyr2542 in bovine Tg), in the bovine Tg structure (red) the α-helix containing the donor Tyr2542 has unwound and is shifted 7 Å away from the thyroxine pocket. (*d*) Tyr24 and Tyr149 in the bovine Tg structure (red) assume similar positions compared with the human Tg structure (gray), although the relative dispositions of the tyrosines and the conformations of the amino-terminal peptide containing the acceptor Tyr24 differ substantially.

Tg is a homodimeric protein packed with multiple Tg type 1 (Tg1) repeating structural motifs. From the N-terminus, which contains three of the observed iodotyrosine residues, a cluster of four Tg1 repeats is joined to another cluster of six Tg1 repeats by a linker domain (Fig. 1 ▸). Each Tg1 repeat contains a characteristic CWCV (Cys–Trp–Cys–Val) sequence motif. The Tg1 cluster is followed by a hinge domain that connects the Tg1 domain to two Tg type 2 (Tg2) repeats spanning from Cys1442 to Cys1513. After the Tg2 region, the Tg type 3 (Tg3) repeat domain forms the arm region of the Tg molecule at one end of a Tg monomer. The Tg3 domain includes the only DIT residue observed in the structure at Tyr2041 (Supplementary Fig. S6*e* ). After the Tg3 domain, the ChEL domain in the C-terminus provides Tg with structural stability and integrity. In our Tg structure, the ChEL domain contains a thyroxinated tyrosine at Tyr2575 and an apparent dehydroalanine at Tyr2542 in close proximity (∼7 Å apart), which suggests that the two form an acceptor–donor pair that couple to synthesize T_4_ at Tyr2575 (Fig. 2 ▸ *d*; discussed further below).

The structure of Tg features many intramolecular disulfide bonds both within and between domains. Each structural domain includes multiple cysteine residues, and a Tg monomer may contain as many as 60 pairs of cysteine residues that are able to form disulfide bonds (Citterio *et al.*, 2018 ▸). In our structure, 60 pairs of opposing cysteine side chains were resolved in each Tg monomer. Nine of the 60 pairs clearly lack disulfide bonds between the opposing cysteine side chains (Supplementary Fig. S7 and Supplementary Table S1), a feature that only becomes apparent at high resolution where individual cysteine rotamers can be unambiguously resolved. To verify that the surprising observation of these nine unbonded cysteine pairs is not caused by disulfide bond breakage due to radiation damage during data collection (Kato *et al.*, 2021 ▸), we calculated per-frame reconstructions using *RELION 3*. The nine pairs of cysteine residues that were observed to not form disulfides in the reconstruction calculated using all movie frames remained unbonded in a reconstruction calculated from only the first frame (data not shown). It remains possible that either these putative linkages are broken even by the relatively minimal (1.4 e^−^ Å^−2^) dose accumulated during the first frame, or that these linkages were irreversibly broken in our starting material, although no reducing agents were used during purification.

### 3.2. Iodotyrosines and hormonogenic sites in bovine Tg   

Our structural model did not yield clear evidence of *de novo* T_3_ formation at the C-terminus of Tg (Citterio *et al.*, 2018 ▸), which occurs within a highly mobile region that was dis­ordered in our reconstruction. Nevertheless, two acceptor–donor pairs were clearly identified in the structure, involving Tyr24 (acceptor)–Tyr149 (donor) and Tyr2542 (donor)–Tyr2575 (acceptor). Our high-resolution cryoEM map and model provide clear structural evidence for Tyr24–Tyr149 and Tyr2575–Tyr2542 functioning as acceptor–donor pairs. Both thyroxine acceptor sites at Tyr24 and Tyr2575 exhibited extra densities in the cryoEM map, revealing two phenolic rings coupled together, forming T_4_ at the site of the acceptor tyrosine (Figs. 2 ▸ *a* and 2 ▸ *b*). Notably, Tyr234 (while being the tyrosine residue in closest proximity to the T_4_ derived from Tyr24) was non-iodinated, indicating that for native bovine thyroglobulin proximity to the thyroxinated tyrosine does not necessarily indicate coupling, but rather may represent a mechanism to ‘dock’ the side chain after T_4_ has formed on Tyr24. Additional amino-acid residues (including serine, threonine and the aforementioned Tyr234) appear to stabilize the iodo moieties on the two phenolic rings of T_4_ (Fig. 2 ▸ *a*). In contrast, Tyr149 exhibited no side-chain density in the cryoEM map, likely indicating the conversion of Tyr149 to dehydro­alanine. Although we cannot eliminate the possibility that this tyrosine side chain is not observed due to positional disorder, there are several reasons to believe that conversion to de­hydroalanine is the explanation for the lack of a side chain at this position. Firstly, the local resolution in the vicinity of this residue is very high, and all other neighboring side chains are well defined. Indeed, the C^β^ atom of Tyr149 itself is well defined, and no density for a phenolic ring is observed even at very low density thresholds. Secondly, although flexible residues with many high-probability rotamers (such as arginine and lysine) quite frequently lack density due to positional disorder, this is much less common for bulky aromatic residues such as tyrosine. Moreover, these first structural data are directly supported by earlier biochemical studies suggesting that Tyr149 is the primary donor for T_4_ formation at Tyr24 of Tg, the most evolutionarily conserved (and primary) site of thyroid hormone synthesis in the human body (Marriq *et al.*, 1991 ▸; Dunn *et al.*, 1998 ▸).

We also note that Tyr2542, the apparent donor and the closest tyrosine residue to the thyroxinated Tyr2575, lacks side-chain density, suggesting that it too has been converted to dehydroalanine. Positively charged amino-acid residues, such as lysine and arginine, were also identified neighboring the thyroxine and DIT sites (Figs. 2 ▸ *a* and 2 ▸ *b*). In the second site that harbors the thyroxinated Tyr2575, serine and lysine residues (Ser2475, Ser2579 and Lys2526) stabilize the T_4_-modified Tyr2575 (Fig. 2 ▸ *b*).

### 3.3. Coupling mechanisms of donor and acceptor tyrosines   

The distance between the Tyr24–Tyr149 acceptor–donor pair is 27.4 Å (C^α^–C^α^) in our structure in which thyroxine has already been formed. Such a distance would not allow direct interaction between the two DIT residues if they were in the same conformation pre-coupling, implying that the donor or acceptor peptide segment must be mobile prior to the coupling reaction. We also carefully reviewed the electron-density maps for the amino-terminal peptide from the cryoEM studies of human Tg (which shows 100% amino-acid identity to the bovine Tg sequence in this region: NIFEYQVDAQ…), in which iodination at Tyr24 had apparently not taken place (Coscia *et al.*, 2020 ▸). This revealed that the non-iodinated amino-terminal portion of Tg has little defined structure and thus is likely to be highly mobile. Compared with the human Tg structure (Coscia *et al.*, 2020 ▸), Tyr24 and Tyr149 in our bovine Tg structure are located in comparable positions, although the conformations of the amino-terminal peptide containing Tyr24 differ substantially (Fig. 2 ▸ *c*). Together, these data suggest that prior to T_4_ formation, the amino-terminal segment containing DIT at Tyr24 must be sufficiently mobile to engage DIT at Tyr149, which is located in the periphery of the Tg molecule and is solvent-exposed. In this scenario, Tyr24 traverses 27 Å to capture the phenolic side chain from the DIT149 donor, thereby forming the outer ring of thyroxine. The peptide bearing thyroxine at Tyr24 then docks in the pocket created by the polar amino-acid side chains noted above (Figs. 2 ▸ *a* and 3 ▸ *a*).

**Figure 3:**
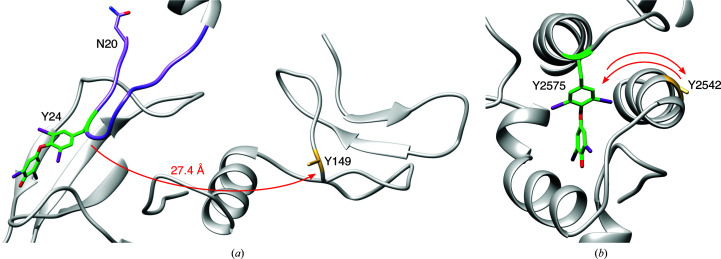
Proposed mechanisms for the coupling of diiodotyrosine side chains at two distinctive sites of Tg. (*a*) The amino-terminal loop (purple) that contains Tyr24 (green) swings by Tyr149 (yellow), where the two rings are coupled to form thyroxine at Tyr24. (*b*) The α-helices that contain Tyr2575 (green) and Tyr2542 (yellow) are brought together to form thyroxine at Tyr2575, and the second helix is displaced to form the binding pocket for T_4_ at Tyr2575.

The second iodotyrosyl coupling site, where the donor Tyr2542 and the acceptor Tyr2575 are located, depicts a similar mechanism in reverse. When compared with the non-iodinated human Tg cryoEM structure (Coscia *et al.*, 2020 ▸), the α-helix with Tyr2542 has been slightly unwound and rotated, resulting in a 7 Å shift from its relative position in non-iodinated Tg, effectively creating the binding pocket for T_4_ at Tyr2575 (Fig. 2 ▸ *d*). Therefore, it would appear that Tyr2542 is the preferred donor to the acceptor Tyr2575, and Tyr2542 then swings out towards the outer environment after completion of the coupling reaction (Fig. 3 ▸ *b*).

## 4. Discussion   

Thyroid hormones are crucial regulators of oxidative metabolism, heart rate, growth and development (Mullur *et al.*, 2014 ▸). The primary thyroid hormone secreted from the thyroid gland is thyroxine. Thyroxine originates from iodinated Tg, the architecture of which has been conserved throughout the vertebrate lineage for the past 500 million years (Citterio *et al.*, 2019 ▸). Tg is a 660 kDa homodimer with a combined total of 140 Tyr residues. At least 37 of the 70 Tyr residues per Tg monomer can be iodinated *in vitro* (Dedieu *et al.*, 2011 ▸), but only a small number of these residues participate in thyroid hormonogenesis (Ogawara *et al.*, 1972 ▸; Izumi & Larsen, 1977 ▸; Xiao *et al.*, 1996 ▸). In our structure of natively iodinated bovine Tg, we have identified two donor–acceptor pairs of Tyr residues involved in intra-monomeric thyroxine formation.

Previous biochemical studies of Tg have utilized Edman degradation, HPLC and mass spectrometry of peptide fragments to pinpoint iodination sites and hormonogenic tyrosine residues in Tg. This has been an extremely successful approach to the identification of DIT acceptors, which represent the sites of hormone formation. However, biochemical approaches have been less definitive in identifying the specific donor partner(s) that yield their iodophenolic side chain to be converted to dehydroalanine in the process of forming thyroid hormone at Tg acceptor sites (Marriq *et al.*, 1991 ▸; Dedieu *et al.*, 2011 ▸; Dunn *et al.*, 1987 ▸; Palumbo, 1987 ▸; Ohmiya *et al.*, 1990 ▸; Palumbo *et al.*, 1990 ▸; Xiao *et al.*, 1996 ▸; Gentile *et al.*, 1997 ▸). In the present study, we examined the structure of iodinated bovine Tg. We report the first visual evidence of formation of thyroxine (T_4_) at Tyr24 and Tyr2575, with conclusive structural evidence of the donor–acceptor pairs.

Recently, with the first report of the cryoEM structure of human Tg, Coscia and coworkers searched within a 15 Å radius as a means to identify the putative donor residues to the Tyr24 acceptor (Coscia *et al.*, 2020 ▸). A review of the cryoEM maps (Coscia *et al.*, 2020 ▸) indicates that the reported human Tg structure studied had no demonstrable iodination, with no detectable MIT, DIT or thyroid hormone residues in the protein. The amino-terminal segment of human Tg appears largely unstructured, but the closest Tyr residue to Tyr24 is Tyr234, and based on this, as well as *in vitro* assays involving iodination of recombinant Tg mutants with lactoperoxidase, Tyr234 was proposed as one of two DIT donors (the other being Tyr149) for thyroxine formation at Tyr24. In our own structure of natively iodinated bovine Tg, we confirm that T_4_ derived from Tyr24 is extremely close to Tyr234 and that the two side chains do directly interact. However, we observe that at native levels of iodination Tyr234 retains its phenolic side chain and is not discernibly iodinated, even when Tyr24 has apparently been completely converted to the T_4_-modified form (Fig. 2 ▸ *a*). Thus, we conclude that at normal levels of iodination Tyr234 is unlikely to serve as a donor to the primary thyroxine formation site at Tyr24 (although it is clearly accessible for modification *in vitro*). Instead, our cryoEM map and atomic model supports the notion that Tyr149 is the DIT donor for thyroxine formation at Tyr24 (Marriq *et al.*, 1991 ▸; Dunn *et al.*, 1998 ▸; Ohmiya *et al.*, 1990 ▸; Palumbo *et al.*, 1990 ▸), as the side chains of Tyr24 and Tyr149 are converted to T_4_ and dehydroalanine, respectively. We cannot exclude the possibility that the situation in human thyroglobulin is different, although the high sequence conservation in this region would argue against this possibility; alternatively, it may be that both sites can be modified under conditions that favor higher iodination levels *in vivo*.

Tyr149 in our structure falls well outside the 15 Å radius searched by Coscia *et al.* (2020 ▸); indeed, we observed that the physical distance between T_4_ at Tyr24 and the dehydroalanine at Tyr149 is >27 Å. One way to explain this observation is that if the donor DIT at Tyr149 is largely immobile, then the unstructured amino-terminal peptide bearing the acceptor at Tyr24 must be mobile prior to its coupling with DIT149. Thus, we propose that the amino-terminal peptide is able to ‘swing’ sufficiently to close the gap separating Tyr24 and Tyr149. Importantly, our model does indicate that the unstructured amino-terminal segment has sufficient flexibility to migrate across this seemingly large distance. Thus, taken together with previously published biochemical studies (Marriq *et al.*, 1991 ▸; Dunn *et al.*, 1998 ▸; Ohmiya *et al.*, 1990 ▸; Palumbo *et al.*, 1990 ▸), we conclude that DIT at Tyr149, but not Tyr234, is likely to be the primary donor for T_4_ formation at Tyr24.

What then, do we make of Tyr234 and its relatively intimate proximity to T_4_ at Tyr24? It has been reported that Tg contains several thyroxine-binding sequences (Benvenga & Guarneri, 2019 ▸). Indeed, we observe that the binding pocket stabilizing T_4_ at Tyr24 includes polar Ser and Thr residues as well as Tyr234 (Figs. 2 ▸ *a* and 3 ▸ *a*). We cannot rule out the possibility that at higher levels of iodination (for example with a high iodide diet) Tyr234 might become iodinated and thus might become available to undergo a coupling reaction with DIT at position Tyr24.

The other acceptor–donor pair we observed in bovine Tg is Tyr2575 and Tyr2542 with modified side chains (Figs. 2 ▸ *b* and 3 ▸ *b*). Both of these Tyr residues have previously been demonstrated to be iodinated upon radiolabeling with ^125^I (Xiao *et al.*, 1996 ▸). Tyr2542 is the closest Tyr residue to Tyr2575, and the two Tyr residues are conserved between human and bovine Tg. Indeed, in the human Tg structure with no iodinated tyrosine side chains, the phenolic rings of the two homologous residues face each other (Coscia *et al.*, 2020 ▸). In iodinated bovine Tg, we observe that the donor dehydro­alanine at Tyr2542 is ∼12 Å from the T_4_ at Tyr2575. However, when compared with the structure of non-iodinated human Tg (Coscia *et al.*, 2020 ▸), the α-helix bearing dehydro­alanine at Tyr2542 is unwound and shifted ∼7 Å away from that of the homologous Tyr residue in human Tg (Fig. 2 ▸ *d*). This suggests that once again significant polypeptide chain movement follows the coupling reaction between the donor and acceptor, and in this case is likely to involve mobility of the donor peptide rather than the acceptor peptide. Specifically, the dehydroalanine at Tyr2542 in our bovine Tg structure faces towards the solvent. Structural evidence suggests that upon iodination and coupling reactions, the α-helix with Tyr2542 shifts away from the T_4_-binding site, which allows T_4_ at Tyr2575 to now interact within a thyroxine-binding pocket (Figs. 2 ▸ *d* and 3 ▸ *b*).

A caveat of this study is the existence of poorly resolved regions in the cryoEM map caused by heterogeneity in post-translational modifications, such as a variable degree of iodination (Fassler *et al.*, 1988 ▸) between individual Tg molecules, as well as a high degree of peptide mobility within certain peptide regions. For example, following the ChEL domain of Tg [which is structurally similar to acetylcholinesterase (MacPhee-Quigley *et al.*, 1986 ▸), comprises the final ∼20% of each Tg monomer sequence and serves to mediate both homodimerization and interaction with upstream Tg domains] at the extreme carboxyl-terminal end is a short unique tail sequence that contains an iodination site yielding the preferential formation of triiodothyronine (Citterio *et al.*, 2018 ▸). This hormone-forming site also appears to be in a peptide segment of high mobility, as it is not visualized in reconstructions of either human Tg (Coscia *et al.*, 2020 ▸) or bovine Tg (this study).

Despite its limitations, the present structure brings us one step closer to understanding the mechanisms of thyroid hormonogenesis. Indeed, this is the first report of a selective set of iodotyrosine side chains resolved in a high-resolution (2.6 Å) structure accounting for thyroid hormonogenesis within Tg. Both of the observed T_4_ residues (Tyr24 and Tyr2575) of Tg are buried in their respective binding pockets in the post-reaction state. Taken with the observed locations of the corresponding donor tyrosine partners (Tyr149 and Tyr2542), this enables us to suggest a detailed coupling mechanism wherein the peptides that contain donor–acceptor pairs exhibit high relative mobility prior to the synthesis of T_4_ and the modified tyrosines are subsequently sequestered in clefts on the surface of Tg.

## Supplementary Material

PDB reference: bovine thyroglobulin, 7n4y

EMDB reference: bovine thyroglobulin, EMD-24181

Supplementary Figures, Supplementary Tables and captions to Supplementary Videos. DOI: 10.1107/S2059798321010056/jb5028sup1.pdf

Supplementary Video S1: CryoET tilt series of a bovine Tg grid without the addition of detergent before grid preparation. DOI: 10.1107/S2059798321010056/jb5028sup2.mp4

Supplementary Video S2: CryoET tilt series of a bovine Tg grid with the addition of 0.2%(w/v) CHAPS before grid preparation. DOI: 10.1107/S2059798321010056/jb5028sup3.mp4

## Figures and Tables

**Table 1:** CryoEM data-collection, refinement and model statistics

Data collection and processing	
Magnification	130 000×
Voltage (kV)	300
Electron exposure (e^−^ Å^−2^)	71.32
Nominal defocus range (µm)	1–2
Pixel size (Å)	1.06
Symmetry imposed	*C*2
Initial particle images	1167730
Final particle images	539260
Map resolution (Å), FSC threshold 0.143	2.61
Map-sharpening *B* factor (Å^2^)	97.7
Refinement	
PDB code	7n4y
EMDB code	EMD-24181
EMPIAR code	EMPIAR-10833
Model composition	
Non-H atoms	37904
Protein residues	2452
R.m.s. deviations	
Bond lengths (Å)	0.017
Bond angles (°)	1.906
Validation	
*MolProbity* score	1.66
Clashscore	5.47
Rotamer outliers (%)	0.15
Ramachandran plot	
Favored (%)	94.66
Allowed (%)	5.34
Outliers (%)	0.00
