# Supplementary material for: The structure of natively iodinated bovine thyroglobulin
Source: Acta Crystallogr D Struct Biol. 2021 Oct 29;77(Pt 11):1451–9. doi: 10.1107/S2059798321010056 (PMC8561740; doi:10.1107/S2059798321010056)
Supplement: Supplementary file 1 [file d-77-01451-sup1.pdf]

STRUCTURAL BIOLOGY

Volume 77 (2021)

Supporting information for article:

The structure of natively iodinated bovine thyroglobulin

Kookjoo Kim, Mykhailo Kopylov, Daija Bobe, Kotaro Kelley, Edward T. Eng, Peter Arvan and Oliver B. Clarke

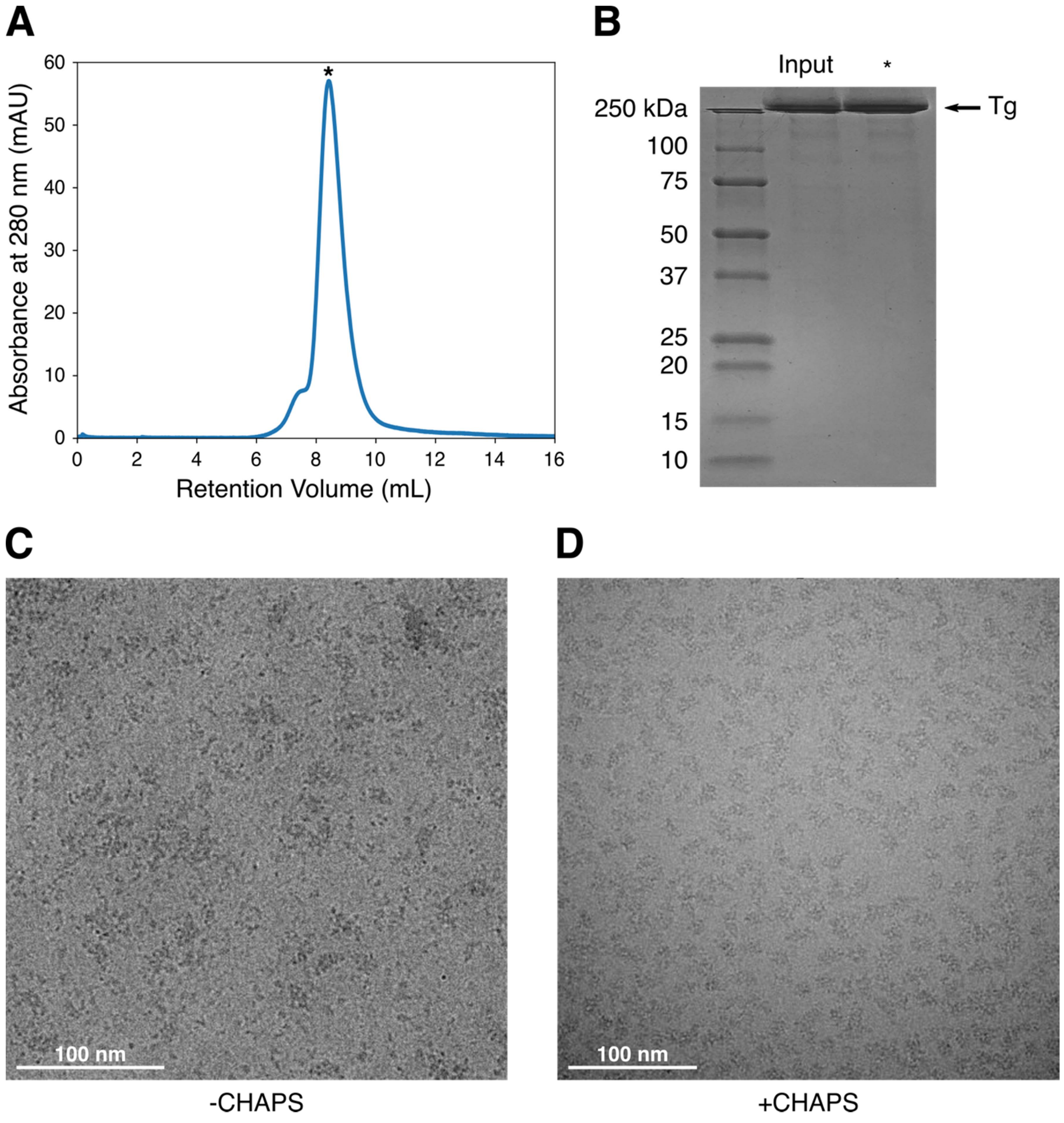

Figure S1 Sample preparation of bovine Tg. **A**: Size exclusion chromatography profile for Tg. The fraction containing Tg (*) was collected for SDS-PAGE analysis and cryoEM grid preparation. **B**: SDS-PAGE gel of the input (Tg in buffer) and the purified Tg fraction. Comparison between Tg samples without 0.2% (w/v) CHAPS addition after SEC **(C)** or with 0.2% CHAPS **(D)**.

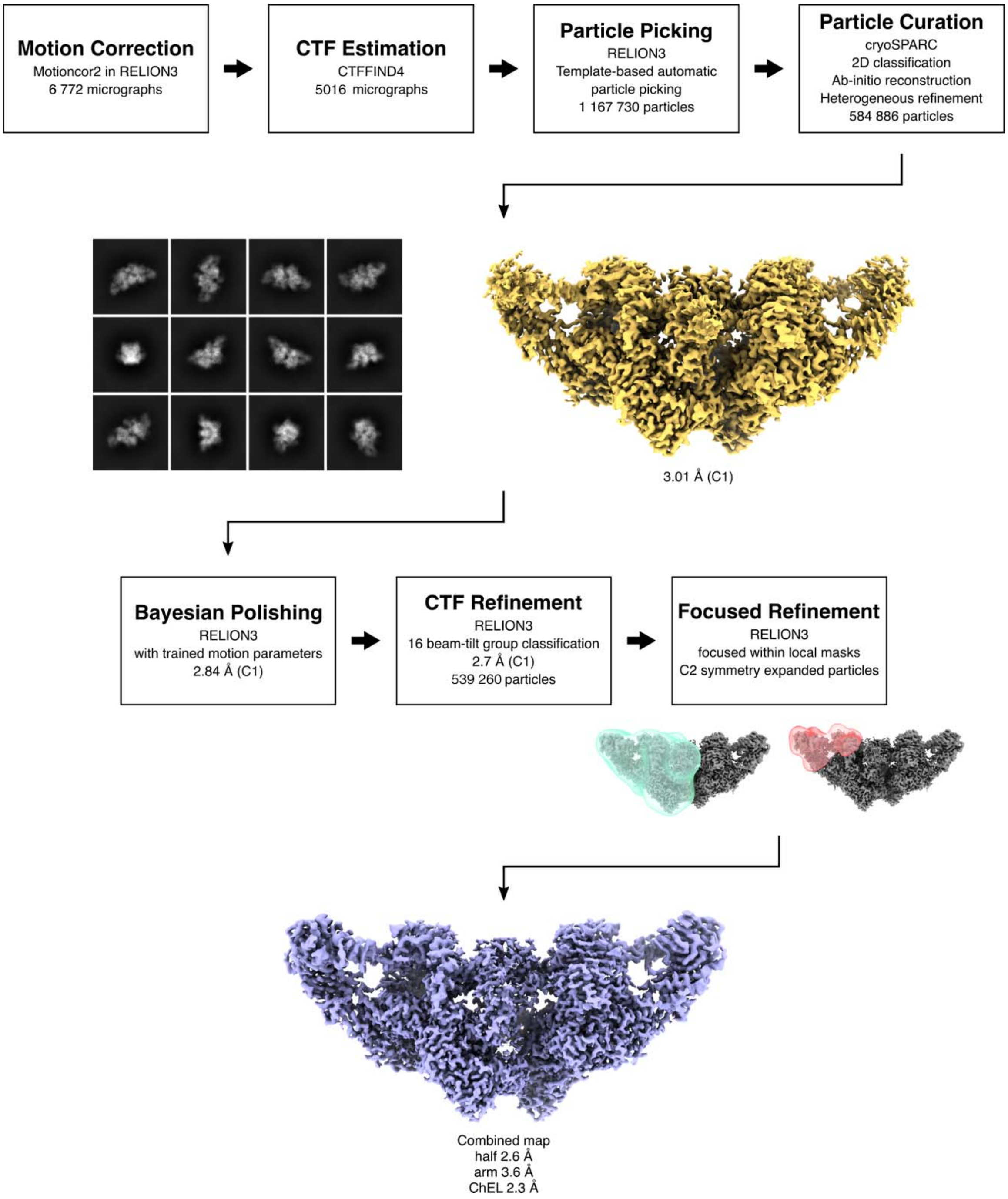

Figure S2 CryoEM data workflow of 3D reconstruction for bovine Tg. The data processing workflow is described in the methods section. Representative 2D classes of 584,886 particles after particle curation is shown. The colored volume densities of 3D reconstructions after key steps are noted by FSC resolutions (0.143 cut-off) and imposed symmetry information. Map contour levels = 0.65 (yellow) & 0.04 (purple). Representations of the local masks surrounding half of the dimeric Tg molecule (turquoise) and the arm region (red) of a monomer are shown.

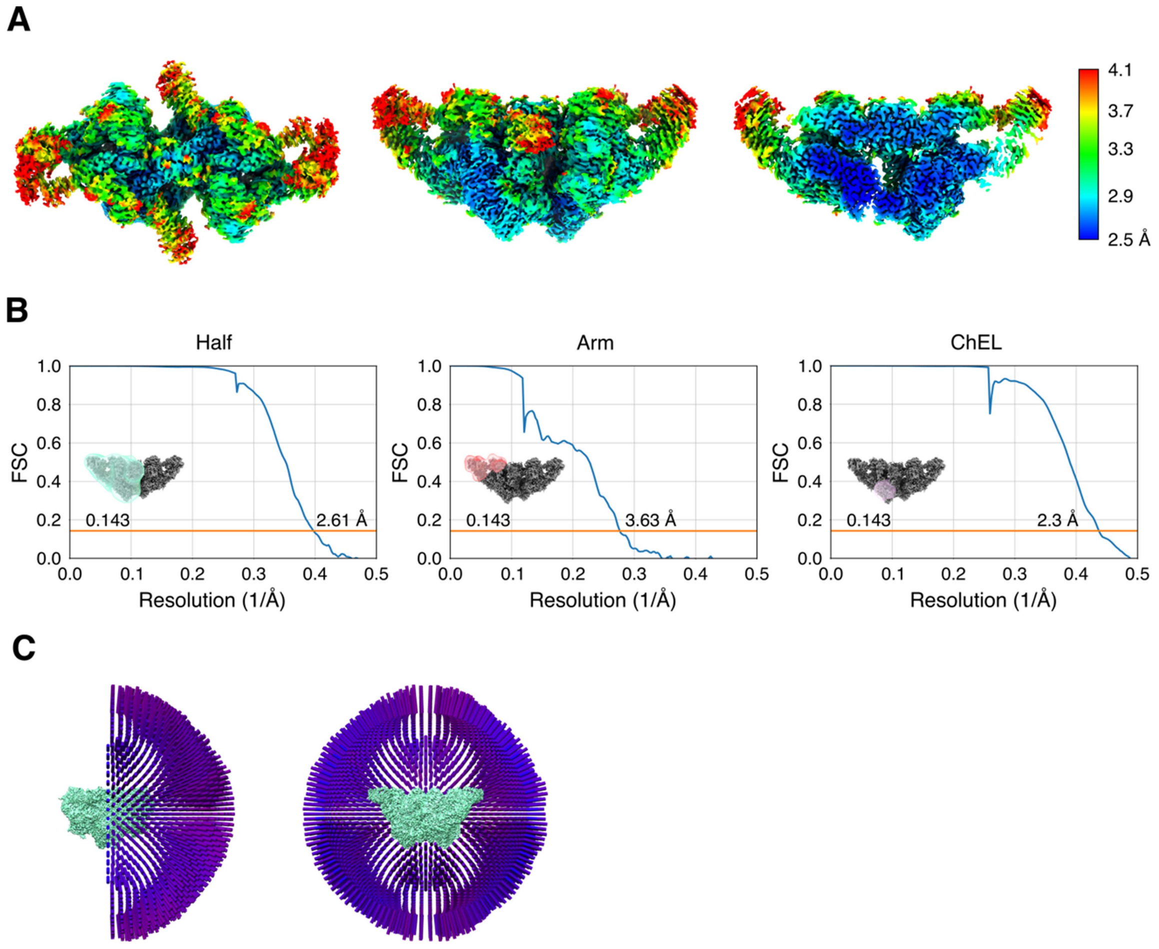

Figure S3 Local resolution distribution, focused 3D refinement, and particle orientations. a) Local resolution plot of the cryoEM map from the global 3D reconstruction. Top view, side view, and one half cutaway to visualize internal density are shown. b**)** Fourier shell correlation plots of cryoEM maps from focused refinements with masks surrounding a half of the dimeric Tg molecule (left), the arm region (middle), and the core ChEL domain (right) with C2 symmetry expansion of particles. c) Particle orientation distribution plot for the 554,003 particles used in the refinement.

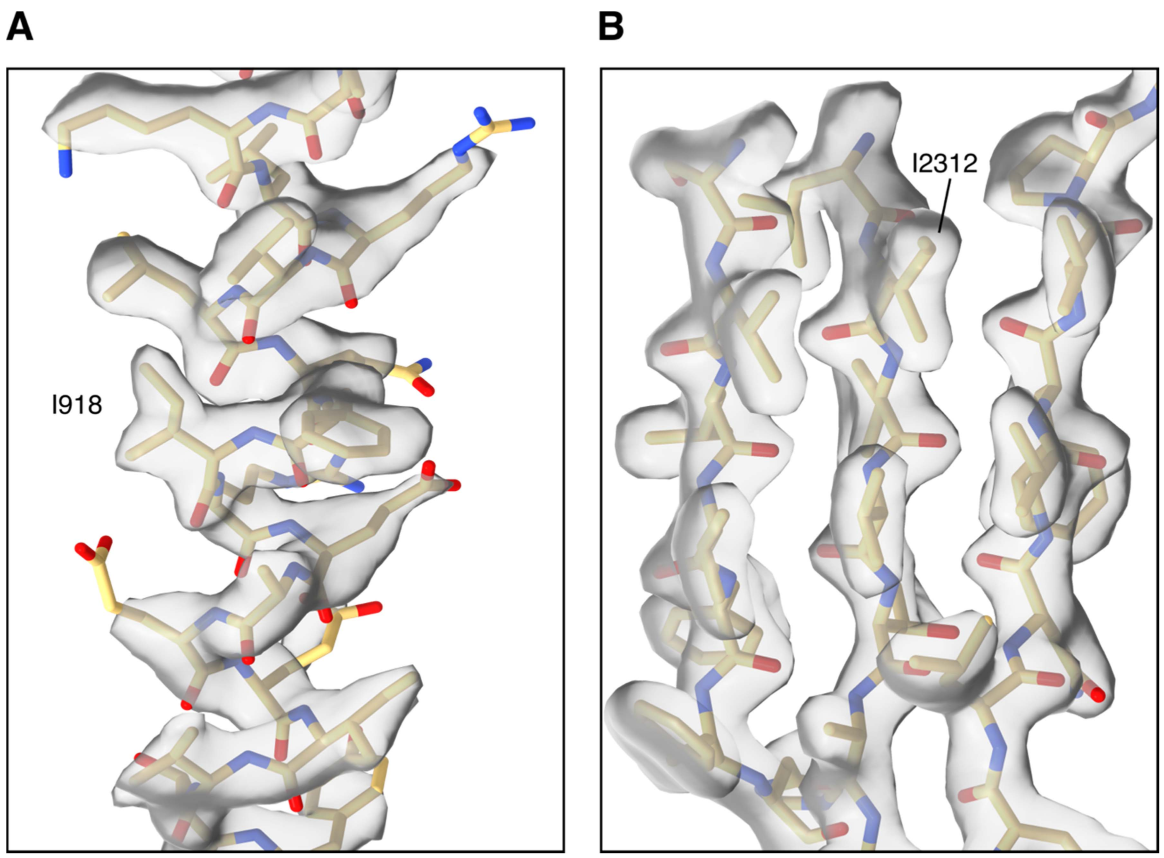

Figure S4 Representative α-helix and β-sheets. Representative model and cryoEM map fit for a typical α-helix around Isoleucine-937 (a) and a typical β-sheet (b). Map contour levels = 0.04 (a) & 0.076 (b).

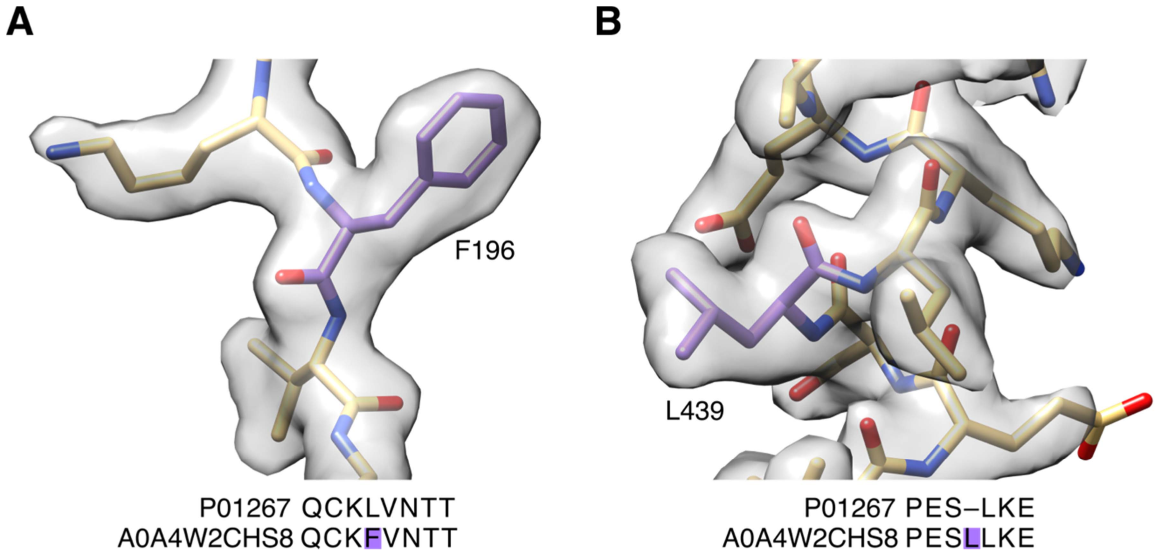

Figure S5 Discrepancies between the bovine Tg structure and the representative amino acid sequence. a) The bovine Tg cryoEM map and model fit around Phenylalanine-196 shows that the resolved structure does not agree with the representative amino acid sequence of bovine Tg (Uniprot P01267). b) Map and model fit around Leucine-439 exhibits an insertion of L439. Two amino acid sequences (Uniprot P01267 and A0A4W2CHS8) for depicted regions were aligned and compared below each panel. The incongruent amino acid residues are colored in purple in the model and the sequence. A0A4W2CHS8 was then used for model building. Map contour level = 0.04

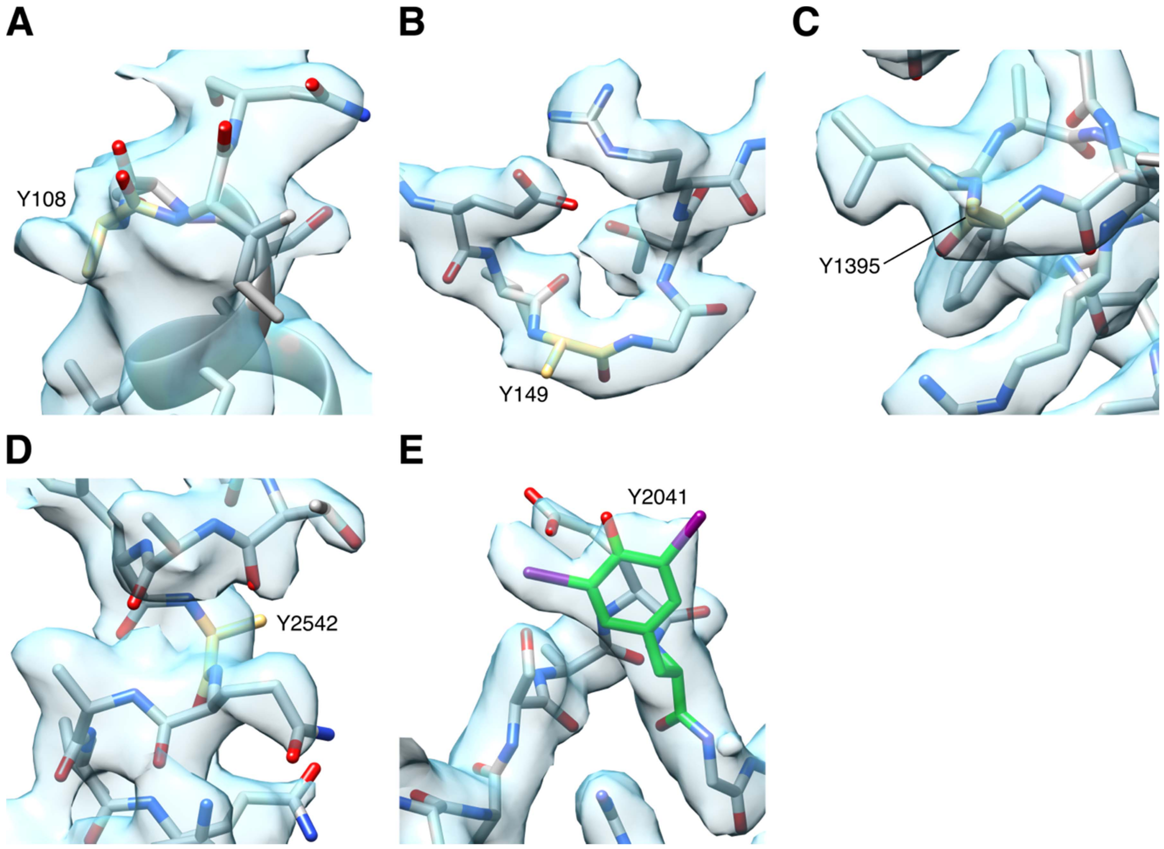

Figure S6 Observed modified tyrosine side chains in the bovine Tg structure and the cryoEM map. Dehydroalanines were assigned based on the observation of lack of tyrosine side chains in the cryoEM map at Y108, Y149, Y1395, and Y2542 (a-d). The map exhibits the di-iodination of the tyrosine side chain at Y2041. Hence, it was assigned as a DIT (e). The assigned dehydroalanines are colored in yellow, and the assigned DIT is colored in green. Map contour levels = 0.03 (a) & 0.034 (b-e).

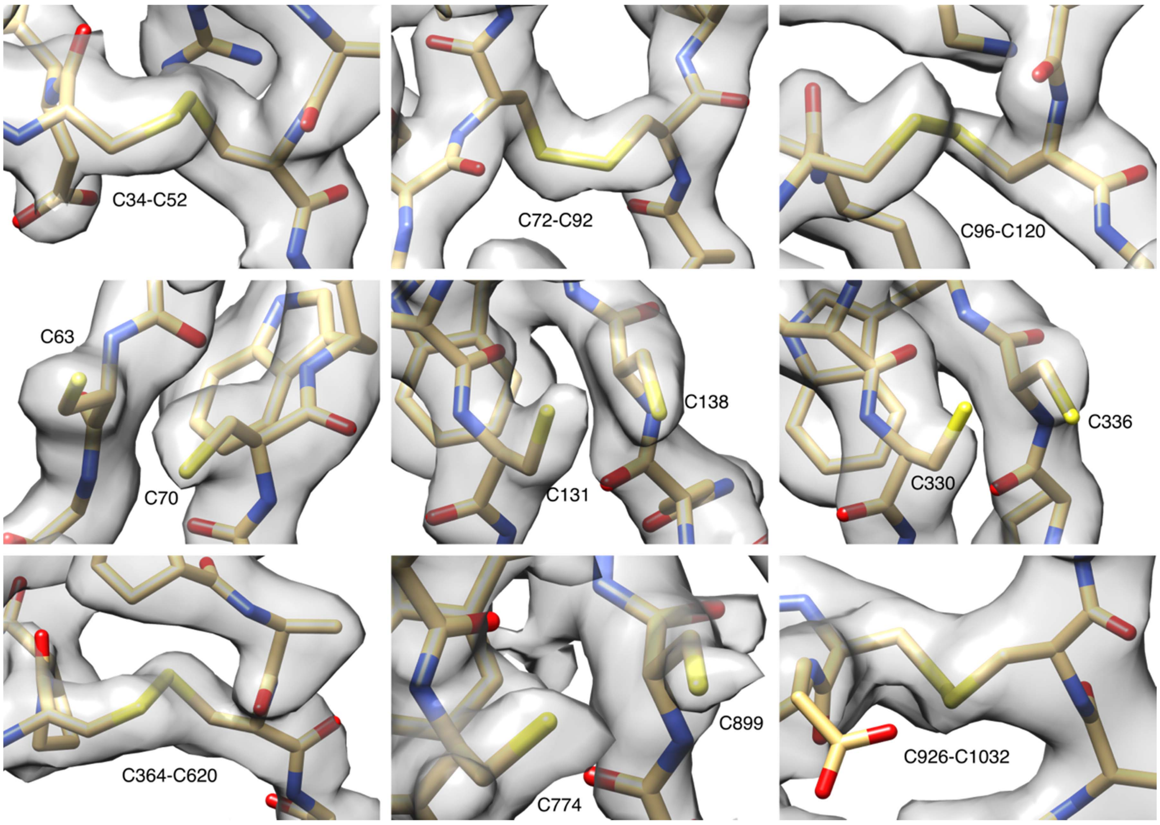

Figure S7 Representative model and cryoEM map fit for opposing cysteine side chains. In addition to the disulfide bonds between two cysteine residues, nine pairs of opposing cysteine side chains without disulfide bonds were resolved in the Tg cryoEM map.

Table S1 Observed opposing cysteine pairs in the Tg structure.

Cysteine Residues	Domain	Notes
34-52	Tg1.1	
63-70	Tg1.1	Disulfide bond is not present.
72-92	Tg1.1	
96-120	Tg1.2	
131-138	Tg1.2	Disulfide bond is not present.
140-160	Tg1.2	
164-183	Tg1.3	
194-235	Tg1.3	
237-297	Tg1.3	
301-319	Tg1.4	
330-336	Tg1.4	Disulfide bond is not present.
338-358	Tg1.4	
364-620	Linker	
408-608	Linker-Tg1.5	Interdomain long-range disulfide bond
631-636	Tg1.5	
638-658	Tg1.5	
662-687	Tg1.6	
698-703	Tg1.6	
705-726	Tg1.6	
730-763	Tg1.7	
774-899	Tg1.7	Disulfide bond is not present.
901-922	Tg1.7	
926-1032	Tg1.7-Tg1.8	Interdomain long-range disulfide bond
1043-1050	Tg1.8	
1052-1074	Tg1.8	
1078-1109	Tg1.9	
1127-1146	Tg1.9	
1150-1170	Tg1.10	
1182-1189	Tg1.10	
1191-1211	Tg1.10	
1216-1265	Hinge	
1232-1246	Hinge	
1250-1282	Hinge	
1332-1348	Hinge	
1442-1462	Tg2.1	
1465-1476	Tg2.1	
1479-1493	Tg2.2	Disulfide bond is not present.
1496-1513	Tg2.2	
1517-1526	Tg1.11	
1546-1568	Tg1.11	
1606-1630	Tg3a.1	Disulfide bond is not present.
1610-1616	Tg3a.1	
1642-1665	Tg3a.1	Disulfide bond is not present.
1727-1752	Tg3b.1	
1731-1737	Tg3b.1	
1736-1837	Tg3b.1	Disulfide bond is not present.
1763-1780	Tg3b.1	
1895-1921	Tg3a.2	
1899-1906	Tg3a.2	
1930-1941	Tg3a.2	
1998-2026	Tg3b.2	
2002-2008	Tg3b.2	
2007-2078	Tg3b.2	
2037-2050	Tg3b.2	
2132-2156	Tg3a.3	
2136-2142	Tg3a.3	
2165-2174	Tg3a.3	
2266-2283	ChEL	
2444-2455	ChEL	Disulfide bond is not present.
2593-2717	ChEL	Long-range disulfide bond

Supplementary Video S1. CryoET tilt series of a bovine Tg grid without the addition of detergent before grid preparation. The black square outlines the region of interest along the z-axis, where the denatured fragments of Tg were observed, suggesting adsorption of Tg particles at the air-water interface.

Supplementary Video S2. CryoET tilt series of a bovine Tg grid with the addition of 0.2% (w/v) CHAPS before grid preparation. Intact Tg particles were observed in the middle of the z-axis, suggesting the addition of the detergent below its critical micelle concentration abolished adsorption and denaturation of Tg particles at the air-water interface.
